# Gastrointestinal metabolites: a potential bridge connecting gut microbiota and myocardial metabolic reprogramming in acute myocardial infarction

**DOI:** 10.3389/fphys.2026.1837224

**Published:** 2026-08-17

**Authors:** Jingxin Zhang, Xiang Xiao, Xuefei Gao, Jiazhen Li, Haoyue Shi, Jing-Yan Han, Yin Li, Lin Li

**Affiliations:** 1 Department of Integration of Chinese and Western Medicine, School of Basic Medical Sciences, Peking University, Beijing, China; 2 Tasly Microcirculation Research Center, Peking University Health Science Center, Beijing, China; 3 The Key Discipline for Integration of Chinese and Western Basic Medicine (Microcirculation), National Administration of Traditional Chinese Medicine, Beijing, China; 4 Key Laboratory of Stasis and Phlegm, State Administration of Traditional Chinese Medicine of the People’s Republic of China, Beijing, China; 5 Beijing Microvascular Institute of Integration of Chinese and Western Medicine, Beijing, China; 6 Department of Integrative Cardiology, China-Japan Friendship Hospital, Beijing, China; 7 Graduate School, Beijing University of Chinese Medicine, Beijing, China; 8 Beijing Hospital of Traditional Chinese Medicine, Capital Medical University, Beijing, China

**Keywords:** acute myocardial infarction, gastrointestinal metabolites, gut microbiota, myocardial metabolic reprogramming, biomarker, non-invasive diagnosis and treatment

## Abstract

**Background:**

Gut microbiota plays a significant role in the occurrence and progression of multisystem disease. Previous studies have indicated that the intestinal microenvironment is closely linked to metabolic and cardiovascular diseases, yet its underlying mechanism remains unclear. We aimed to explore the association between the gut microbiota-metabolite network and cardiovascular function with the transcriptomic changes in the myocardium of acute myocardial infarction (AMI) mice.

**Methods:**

Eight-week-old C57BL/6J mice were adaptively fed for 7 days and then divided into the AMI model group and Sham group. After surgery, multiple approaches were used to assess the physiological and pathological changes in mice. At 7 days post-surgery, cardiac sections were analyzed using H&E staining and Masson staining. The gastrointestinal contents collected from the AMI and Sham group mice were analyzed by 16S rRNA sequencing and untargeted metabolomics and apical myocardial tissues were harvested for transcriptomic detection. followed by multi-omics association analysis between the differential gut microbiota and metabolites. Taking metabolites as the bridge, an integrated multi-omics correlation analysis was conducted.

**Results:**

Compared with the Sham group, mice in the AMI group exhibited obvious myocardial pathological damage and significant depletion of gut microbiota such as *Christensenellaceae_R-7_group*, *Allobaculum*, and *Akkermansia*. These changes may be associated with regulation of pathways such as retrograde endocannabinoid signaling, glycerophospholipid metabolism, riboflavin metabolism and primary bile acid biosynthesis, and metabolites such as nitrogen/aromatic metabolites, phospholipid metabolites, and oxidative stress related metabolites. Myocardial transcriptomic results revealed that genes such as *Sdha*, *Hadha* and *Hsdl2* were significantly downregulated in the AMI group, and the DEGs were highly enriched in pathways including fatty acid β-oxidation, mitochondrial respiratory chain and ATP metabolism, suggesting severe energy metabolism disorder in the myocardium of AMI mice.

**Conclusions:**

Myocardial injury in AMI may show a link between the remodeling of gut microbial structure. Correlative correlations indicate that altered gastrointestinal metabolites, as products of gut microbiota, are closely associated with myocardial metabolic reprogramming after AMI, whereas causal effects remain to be verified by further functional experiments.

## 1 Introduction

Acute myocardial infarction (AMI) is characterized by irreversible myocardial necrosis from coronary plaque disruption and blood flow interruption (Nabel and Braunwald, 2012), and is one of the most prevalent and incident CVDs globally (Henein et al., 2022). It is often associated with complications such as malignant arrhythmias and cardiogenic shock (Anderson and Morrow, 2017), and may progress to heart failure or sudden cardiac death (Anderson and Morrow, 2017; Curtain et al., 2024). Therefore, CVDs, including AMI are significant health and socioeconomic burdens (Alnemer, 2024; Kilpi et al., 2017; Plakht et al., 2023). Therefore, there is an urgent need for novel pre-onset non-invasive diagnostic biomarkers for early disease identification.

Systemic inflammation and energy metabolism disorder are the key underlying mechanisms of AMI (Nabel and Braunwald, 2012; Torp et al., 2023). Gut microbiota and metabolites could influence AMI physiology and pathology via metabolic signaling and energy substrate regulation (Snelson et al., 2025; Rivera et al., 2024; Ahlawat et al., 2021). For instance, gut microbiota could interact with the host immune system to activate systemic inflammation and imbalance of energy metabolism, thereby exacerbating myocardial ischemia-reperfusion (I/R) injury and promotes AMI progression (Tang et al., 2024). Besides, gut microbiota significantly modulating cholesterol metabolism and bile acid production (Kazemian et al., 2020), gut microbiota imbalance triggers metabolic abnormalities (Libby, 2017). Furthermore, the digestive system is the initial site of lipid metabolism and maintains lipid homeostasis by regulating dietary and endogenous lipid balance (Kochkarian et al., 2025). Targeting gut microbiota and metabolites can alleviate myocardial mitochondrial dysfunction and oxidative stress (Luo et al., 2025; Brunt et al., 2020). Therefore, gut microbiota and metabolites may serve as potential non-invasive candidate therapeutic targets.

However, the precise molecular mechanisms underlying the organ crosstalk are unclear. Therefore, our study explored potential gut-heart axis pathways by collecting gastrointestinal contents and myocardial tissue samples from the Sham and AMI group mice to analyze the correlations among 16S rRNA sequencing, untargeted metabolomics, and transcriptomics data using bioinformatics tools. We aimed to provide a theoretical basis for the prevention, diagnosis, and treatment of AMI targeting gastrointestinal contents, and to suggest the feasibility of developing non-invasive cardiovascular disease (CVD) management strategies based on the gastrointestinal health.

## 2 Methods

### 2.1 Establishment of acute myocardial infarction model mice

Eight-week-old male C57BL/6 mice were divided into the Sham group and the AMI group (n = 6 per group). The randomization process was performed by an independent researcher who was not involved in data collection or analysis. Mice in each group were then weighed, anesthetized, and fixed in the supine position, followed by depilation, skin preparation, and disinfection. The limbs and head were fixed, and electrocardiography was performed from standard limb leads II using LabChart 8. Subsequently, the skin over the precordial region was incised and the muscles were bluntly dissected. The chest wall was opened at the 3^rd^-4^th^ intercostal space and the heart was then compressed outside the chest manually. In the AMI group mice, the left anterior descending coronary artery was ligated with a 6–0 silk suture needle, 3–4 mm below the inferior edge of the left atrial appendage. Successful ligation was indicated by blanching of the myocardial tissue below the ligation site. Mice in the Sham group underwent thoracotomy without ligation of the left anterior descending coronary artery. The chest cavity was then closed, and the chest was squeezed to eliminate intrathoracic air. The mice were placed on a warm pad at 40°C to recover from anesthesia. Tissue samples were collected on the 7th day after AMI modeling. Before sampling, mice in each group were weighed. The lesioned cardiac tissue above the apex (half of the sample) was harvested for staining, and the remaining half of the sample was used for transcriptome sequencing. Mouse fecal samples were collected for 16S RNA sequencing and untargeted metabolomics. Animal experiments were conducted according to the guidelines of the Experimental Animal Center and were approved by the Medicine Experimental Animal Research Ethics Committee of the Peking University Health Science Center (Approval No. PUIRB-LA2022590).

### 2.2 H&E and masson staining of myocardial tissue

The lesioned cardiac tissue above the apex (1/2) was fixed in 4% paraformaldehyde solution, followed by dehydration, embedding, and sectioning. After baking and dewaxing, the myocardial tissue sections were hydrated using a graded ethanol series. For H&E staining, the sections were stained with hematoxylin for 5 min followed by eosin for 5–10 seconds. For Masson staining, the sections were sequentially incubated in Weigert’s iron hematoxylin staining solution (8 min), acid ethanol differentiation solution (15 s), Masson bluing solution (5 min), ponceau fuchsin staining solution (1 min), weak acid working solution (30 s), phosphomolybdic acid solution (1 min), and aniline blue staining solution (2 min). They were then rinsed and differentiated in three consecutive buckets of 1% glacial acetic acid (8 s per bucket), followed by dehydration with a graded ethanol series, clearing with xylene, and mounting with neutral balsam.

### 2.3 16S rRNA sequencing

Bacterial diversity was identified using primers targeting the 16S V4 region (515F and 806R primers). Total microbial RNA was extracted from mouse fecal samples using MolPure^®^ Magnetic Universal Viral DNA/RNA Kit (18521ES Yeasen Biotechnology Co., Ltd., Shanghai, China). The extracted RNA was then subjected to a one-step PCR amplification with 30 cycles using 15 µL Phusion^®^ High-Fidelity PCR Master Mix (New England Biolabs) and 0.2 µM primers. The qualified PCR products were purified using magnetic beads, quantified by enzyme-linked immunosorbent assay, and mixed in equal amounts according to their concentrations. The PCR products were detected by electrophoresis on a 2% agarose gel, and the target bands were recovered. After constructing sequencing libraries, they were quantified using a Qubit fluorometer and q-PCR. After passing quality control, the libraries were sequenced on a NovaSeq 6000 platform with PE250 paired-end reads.

### 2.4 Preprocessing of 16S rRNA sequencing data

The sample-specific data were demultiplexed from the raw sequencing data based on the barcode sequences and PCR amplification primer sequences. After truncating the barcode and primer sequences, FLASH software (Version 1.2.11) (Magoc and Salzberg, 2011) was used to assemble the reads for each sample and generate the raw Tags data (RawTags). Subsequently, Cutadapt and fastp (Version 0.23.1) software tools were used for data assembly and filtering (Bokulich et al., 2013). Chimeric sequences were removed by comparing with the species annotation databases [Silva database (https://www.arb-silva.de/) for 16S] to obtain high quality sequence reads (Effective Tags) for final analysis (Edgar et al., 2011). DADA2 module in the QIIME2 software (Version QIIME2-202202) was used for denoising and obtain the final Amplicon Sequence Variants (ASVs) and feature tables (Edgar et al., 2011). Species annotation was performed with the QIIME2 software using the Silva 138.1 database.

### 2.5 Alpha and beta diversity analysis

To evaluate the microbial diversity within and between different treatment groups of mice, alpha and beta diversity analyses were conducted. First, 16S rRNA sequencing data was used to confirm the diversity and abundance of species in various samples and groups. Then, the QIIME2 software was used to calculate the Shannon, Simpson, Chao1, and Pielou_e indices for evaluating alpha diversity. Perl was used to calculate weighted and unweighted Unifrac beta diversity distances and generate heatmaps. A UPGMA clustering tree was constructed based on the weighted Unifrac distance matrix using the UPGMA.tre function in QIIME. Principal Coordinate Analysis (PCoA) and Non-Metric Multidimensional Scaling (NMDS) were performed using the R software with the ade4 and ggplot2 packages.

### 2.6 Differential analysis of gut microbiota composition at various levels and functional prediction

Differences in microbial community composition between the two groups were analyzed at the phylum, genus, and species taxonomic levels. Multiple statistical algorithms, including Anosim, Adonis, MRPP, Simper, Student’s t-test, MetagenomeSeq, and LEfSe, were implemented to characterize structural divergence of gut microbiota. PICRUSt2 (Version 2.3.0) was utilized to predict metagenomic functional profiles from marker gene sequences.

The Benjamini–Hochberg (BH)/the false discovery rate (FDR) correction was uniformly applied to all differential analyses, covering microbiota statistics at the phylum/genus/species tiers, metabolomic KEGG enrichment, as well as transcriptomic GO and KEGG enrichment. For all statistical tests, raw *P-values* were corrected via the BH procedure to generate adjusted *P-values (P-adjust)*. Significance was strictly defined as *P* < 0.05. All taxa, metabolites, and enriched pathways marked as statistically significant in all figures meet this FDR threshold. Any exploratory analyses performed without FDR correction are clearly labeled with raw unadjusted *P* in the corresponding figure legends.

### 2.7 Untargeted metabolomics sequencing

Fecal samples (100 mg) from each group of mice were weighed, ground in liquid nitrogen, mixed with 500 μL of 80% methanol, and incubated at room temperature for 10 min. The extracts were frozen at -20°C overnight. Then, they were centrifuged at 15000 g for 20 min at 4°C. The supernatant (metabolite extract) was injected into LC-MS for analysis. Equal volumes of each experimental sample were mixed to prepare the quality control (QC) samples.

Raw mass spectrometry data was converted to mzXML format using ProteoWizard software. Then, it was processed with the XCMS software for peak extraction, alignment, and correction of retention time and total peak area. Peaks with a missing rate greater than 50% were filtered out. Based on the corrected and filtered mass spectrometry data, the Novogene database was used to identify the metabolites. Secondary annotation of metabolite ions was performed to confirm the identification and perform quantitative analysis of the metabolites. Candidate identifiers were annotated using the Human Metabolome Database (HMDB, https://hmdb.ca) and Kyoto Encyclopedia of Genes and Genomes (KEGG) pathway database (https://www.kegg.jp) to determine the physicochemical properties and biological functions of metabolites, respectively. Subsequent multivariate statistical analyses of metabolites were performed using the Principal Component Analysis (PCA) and Partial Least Squares-Discriminant Analysis (PLS-DA) to determine the differences in metabolic patterns between different groups. Hierarchical Cluster Analysis (HCA) and metabolite correlation analysis were performed to determine the relationships between samples and metabolites. Furthermore, metabolic pathway analysis was performed to deduce the biological significance of the metabolites.

### 2.8 Transcriptomics sequencing

Total RNA was extracted from the apical myocardial tissues of mice in the AMI group and Sham group using Trizol reagent (Invitrogen, California, USA). After strictly detecting the purity, concentration and integrity of RNA, the qualified samples were sent to Novogene Co., Ltd.(Beijing, China) for transcriptome sequencing and bioinformatics analysis in accordance with its standard procedures.

Referring to Novogene’s protocol, ribosomal RNA (rRNA) was removed using the Ribo-Zero™ kit, and strand-specific libraries were constructed with the NEBNext^®^ kit. Fragments of 150~200 bp were selected and purified by PCR to obtain high-quality libraries; library quality control was completed by Qubit 2.0, Agilent 2100 and qRT-PCR (effective concentration > 2 nM).

Sequencing was performed on the Novogene Illumina Novaseq platform using the PE150 mode. Raw data were filtered through its self-developed pipeline to obtain clean reads; after aligning to the mouse reference genome (mm10), differentially expressed genes (DEGs) between the AMI group and Sham group were screened using DESeq2 software (|log2FC| > 1 and adjusted P-value < 0.05), followed by Gene Ontology (GO) and KEGG enrichment analyses.

### 2.9 Multi-omics correlation analysis of gastrointestinal microbiota and metabolites

The sequencing data was first analyzed to evaluate the expression of key microbiota and metabolites. Pearson correlation analysis was performed to calculate the correlation coefficients (r) and *P values* based on the relative abundances of all the differential gut microbia at the genus level and the quantitative values of all the differentially expressed metabolites between the two groups of mice using *P* < 0.05 and |r| > 0.5 as threshold parameters. Normality testing (Shapiro-Wilk test) was performed on all normalized relative abundance data of gut genera and standardized metabolite/gene expression values; the majority of datasets conformed to normal distribution, satisfying the prerequisite of Pearson correlation for linear correlation assessment. Heatmaps were generated using the R corrplot package. Scatter plots were generated using the R ggplot package. Chord diagrams and Sankey diagrams were generated using the R circlize package. Network diagrams were generated using the mixOmics package in R language.

### 2.10 Multi-omics correlation analysis of DEGs in myocardial tissue and gastrointestinal metabolites

For the association analysis of metabolome and transcriptome, Pearson correlation analysis was performed to calculate correlation coefficients (r) and *P values* between differential genes and differential metabolites, with Top 50 differential genes and metabolites (sorted by p value) displayed, and *P* < 0.05 marked with *. Heatmaps were generated using R corrplot package, and correlation networks were plotted with R mixOmics package. KEGG enrichment analysis was conducted to identify common pathways, and bubble plots were drawn via R ggplot2 package; common pathways were visualized by iPath and pathview packages in R.

### 2.11 Statistical analysis

Bioinformatics analysis was performed using the R software. GraphPad Prism 9.0 software was used to analyze all experimental measurement data. The data were expressed as mean ± standard deviation (SD). The t-test was used for pairwise comparison when the data conformed to a normal distribution. For data with a non-normal distribution, Wilcoxon rank-sum test was used for pairwise comparison, and Bonferroni method was used for inter-group correction. *P value* < 0.05 was considered as statistically significant.

## 3 Results

### 3.1 Characteristics of myocardial pathology in the AMI model mice

**Figure 1:**
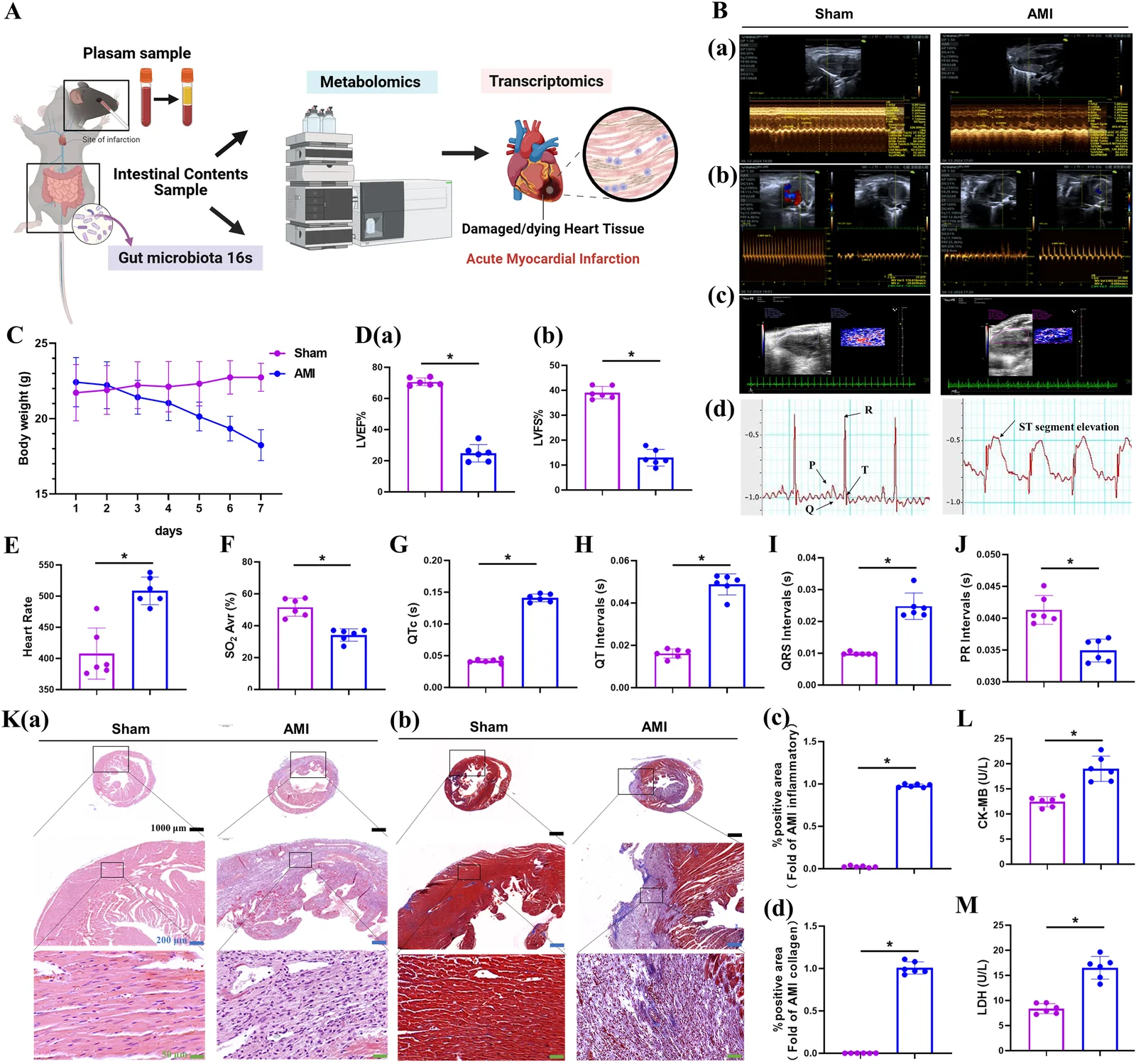
AMI group mice exhibit decreased cardiac function, myocardial inflammation, and fibrosis. **(A)** Schematic overview of the multi-omics study design in a mouse model of AMI; **(B) (a-b)** Representative echocardiography; **(c)** Photoacoustic imaging of the left ventricular anterior wall; **(d)** Representative electrocardiogram (ECG); **(C)** Changes in the body weight at 7 days after surgery; **(D)** (a) Left ventricular ejection fraction (LVEF%); **(b)** Left ventricular fractional shortening (LVFS%) **(E)** Heart rate (HR); **(F)** Blood oxygen content in the left ventricular anterior wall; **(G-J)** QTc, QT intervals, QRS intervals, and PR intervals; **(K) (a)** H&E-staining; **(b)** Masson staining (scale bars = 1000 μm, 200 μm, and 50 μm); **(c-d)** Inflammatory infiltration and collagen deposition analysis; **(L-M)** Serum CK-MB and LDH level. * *p* < 0.05 vs. Sham; 6 mice per group; Error bars represent standard deviation.

We established a mouse model of AMI, and collected plasma, damaged or dying heart tissue, and gut intestinal contents for multi-omics analysis (**Figure 1A**). The AMI group of mice showed impaired cardiac function, accompanied by myocardial inflammation and fibrosis. The AMI group mice showed a significant weight loss trend compared to the Sham group mice (**Figure 1C**). Echocardiography results showed significant left ventricular dysfunction in the AMI group mice [**Figure 1B (a, b)**]. Compared with the Sham group, the AMI group mice showed significantly reduced left ventricular ejection fraction (EF) and left ventricular fractional shortening (FS) (**Figure 1D**) and significantly increased heart rate (HR) (**Figure 1E**). Cardiac photoacoustic imaging showed changes in the hemodynamics of the left ventricular anterior wall [**Figure 1B (c)**] and significantly decreased blood oxygen levels in the AMI group mice (**Figure 1F**). Electrocardiography results showed altered cardiac conduction function with a marked elevation of the ST segment in the AMI group mice [**Figure 1B (d)**]. Compared with the Sham group, the AMI group mice showed significantly prolonged QTc, QT, and QRS intervals (**Figures 1G–I**) and shortened PR interval (**Figure 1J**). Furthermore, significant morphological changes appeared in the myocardial tissues of the AMI mice [**Figure 1K (a, b)**]. Compared with the Sham group, the myocardial tissues of the AMI group exhibited extensive pathological changes, including disordered myocardial fiber structure, scattered hemorrhage and necrosis, inflammatory cell infiltration [**Figure 1K (c)**], and collagen deposition [**Figure 1K (d)**]. Furthermore, the serum activities of myocardial injury markers, CK-MB and LDH, were significantly higher in the AMI group mice compared with the Sham group mice (**Figures 1L, M**). These results demonstrated that the mouse AMI model established by ligation of the left anterior descending coronary artery was successful.

### 3.2 Changes in gut microbiota in the AMI group mice

**Figure 2:**
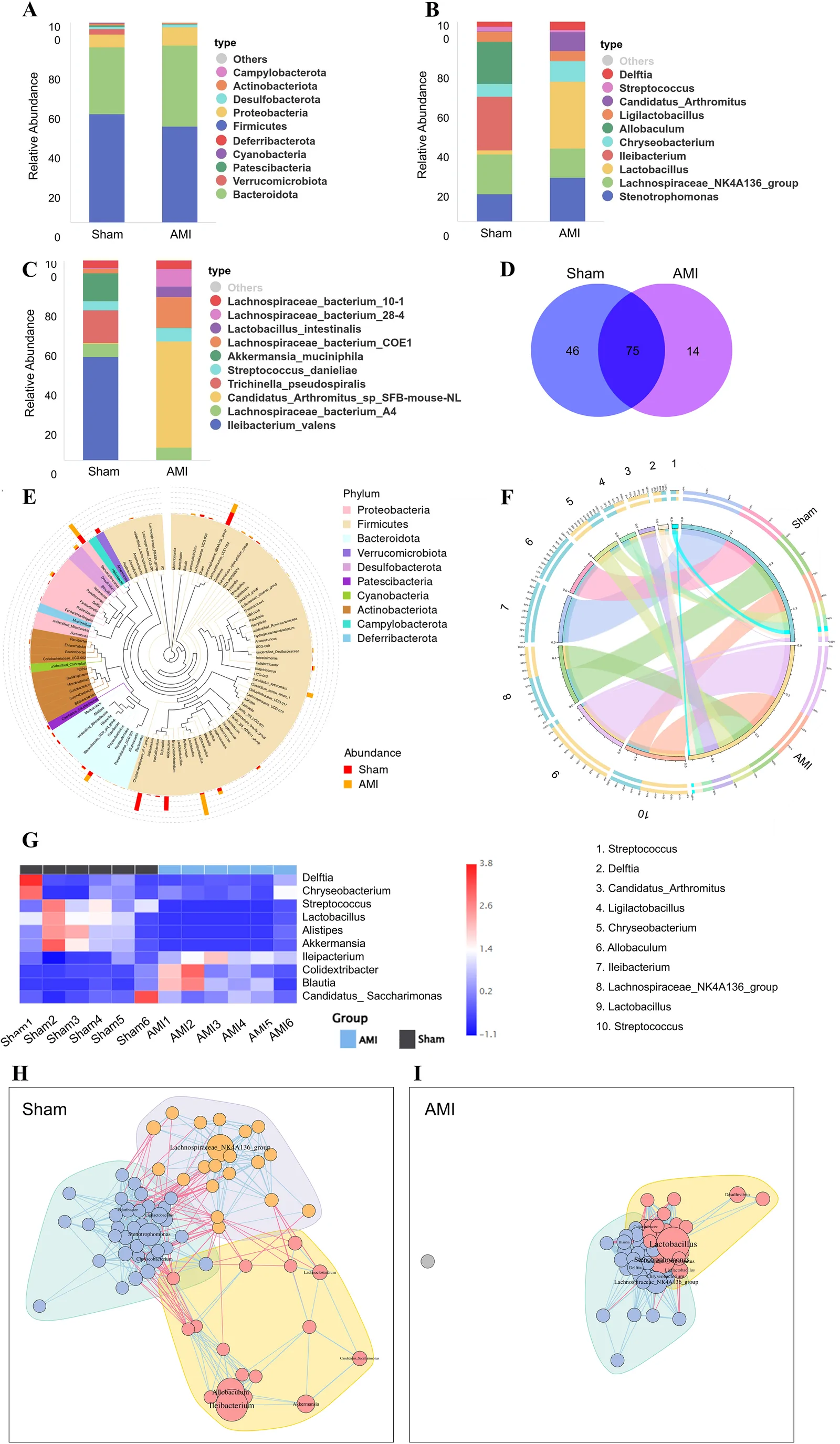
The AMI group mice show significant alterations in the composition of the gut microbiota. **(A-C)** Differences in the gut microbiota between the AMI and Sham group mice at the phylum, genus, and species levels; **(D)** Venn diagram shows the number of shared, unique, and depleted ASVs between the AMI and Sham groups of mice at the genus level; **(E)** Phylogenetic tree of gut microbiota at the genus level in the AMI and Sham group of mice; **(F)** Circos plot shows the sample-gut microbiota species relationships at the genus level in the AMI and Sham group of mice; **(G)** Clustering heatmap shows relative abundances of the gut microbial genera in the AMI and Sham group of mice. **(H, I)** MetaNet species modular association network diagram of gut microbiota; The node represents a genus, and the size of the node indicates the relative abundance of the genus. The edge represents the functional-based positive or negative association between genera, with red edges indicating positive correlation and blue edges indicating negative correlation. The overall network topology coefficients are shown in **Supplementary Table 1**.

Sequencing analysis of the V4 region of the fecal 16S rRNA gene was performed to determine alterations in the gut microbiome of the AMI mice. Compared with the Sham group, the AMI group mice exhibited significant differences in the relative abundances of gut microbiota at the phylum, genus, and species levels (**Figures 2A–C**).

At the phylum level, Firmicutes and Bacteroidetes were the most predominant phyla in both the Sham and AMI groups, while the ratio of them was unusual in this model (Zhong et al., 2023; Wang et al., 2025) due to the disease progression or sustained intestinal epithelial damage. The abundance of Firmicutes in the AMI group (48.14%) was significantly lower than in the Sham group (54.46%). In contrast, compared to the Sham group, the AMI group mice showed significantly higher abundances of Bacteroidetes (40.70% vs. 33.46%) and Proteobacteria (9.03% vs. 6.42%). Furthermore, Verrucomicrobiota were mostly undetectable in the AMI group but present at a low level in the Sham group (0.00% vs. 2.64%) (**Figure 2A**).

At the genus level, compared to the Sham group, the AMI group mice demonstrated significantly higher abundances of *Stenotrophomonas* (7.65% vs. 5.03%), *Lactobacillus* (11.65% vs. 0.74%), *Chryseobacterium* (3.56% vs. 2.33%), *Candidatus_Arthromitus* (3.30% vs. 0.11%), and *Delftia* (1.82% vs. 0.32%), as well as lower abundances of *Lachnospiraceae_NK4A135_group* (5.06% vs. 7.30%) and *Streptococcus* (0.87% vs 0.43%). Moreover, *Ileibacterium* and *Allobaculum* were rarely detected in the AMI group, but were present in the Sham group (9.84% and 7.75%) (**Figure 2B**). Previous studies have shown that *Streptococcus* and *Bacteroides* are associated with systemic inflammation and AMI (Zhou et al., 2018; Kwun et al., 2020), which was consistent with our results. Besides, 75 amplicon sequence variants (ASVs) were shared between the AMI group and the Sham group samples. Furthermore, 14 unique ASVs were detected and 46 ASVs were significantly depleted or undetectable in the AMI group compared to the Sham group (**Figure 2D**).

At the species level, compared to the Sham group, the AMI group mice showed high relative abundances of *Candidatus Arthromitus* sp. SFB-mouse-NL (3.30% vs. 0.11%), *Lachnospiraceae bacterium* COE1 (0.96% vs. 0.45%), *Lactobacillus intestinalis* (0.32% vs. 0.00%), and *Lachnospiraceae* bacterium 28_4 (0.54% vs. 0.07%). In contrast, the relative abundances of *Ileibacterium valens, Akkermansia muciniphila*, and *Ticchinella pseudospiralis* were significantly lower in the AMI group mice compared to the Sham group (**Figure 2C**).

To further investigate the phylogenetic relationships of genera, representative sequences of the top 100 genera (mean relative abundance > 0.01%) were aligned for the construction of a genus-level phylogenetic tree (**Figure 2E**). Additionally, the top 10 taxa by average relative abundance were screened at the phylum, genus, and species levels. Circos plots were used to visualize the taxon-sample associations with |r| > 0.5 (**Figure 2F**; **Supplementary Figures 1A, B**), and hierarchical clustering heatmaps based on the Bray-Curtis dissimilarity index were plotted to show the intergroup variation in taxon abundance (**Figure 2G**; **Supplementary Figures 1C, D**).

MetaNet species modular association network analysis aims to clearly and intuitively demonstrate the interaction patterns of distinct species in biological systems, thereby mining the biological significance of the species abundance data (**Figures 2H, I**). The differences in the community structure between the two groups of microbiotas were clearly quantified, enabling objective comparison of the microbiota structures. Based on the topological coefficients such as modularity and average path length (**Supplementary Table 1**), the microbiota in the AMI group showed poorer stability, lower metabolic efficiency, and incomplete anti-interference ability and functional coordination compared with the Sham group. The core functional modules were identified through degree centrality, core module proportion, and other indicators, thereby providing targets for subsequent microbiota interventions such as prebiotic supplementation and functional module repair.

These findings demonstrated significant gut microbiota dysbiosis in the AMI group mice, characterized by the enrichment of *Bacteroides* and *Proteobacteria*, as well as depletion of *Verrucomicrobiota, Ileibacterium*, and *Allobaculum*. Our data showed that *A. muciniphila* was significantly depleted in the AMI group. Therefore, gut microbial dysbiosis may contribute to the pathophysiological mechanisms underlying AMI.

### 3.3 Analysis of α-diversity and β-diversity of gut microbiota

**Figure 3:**
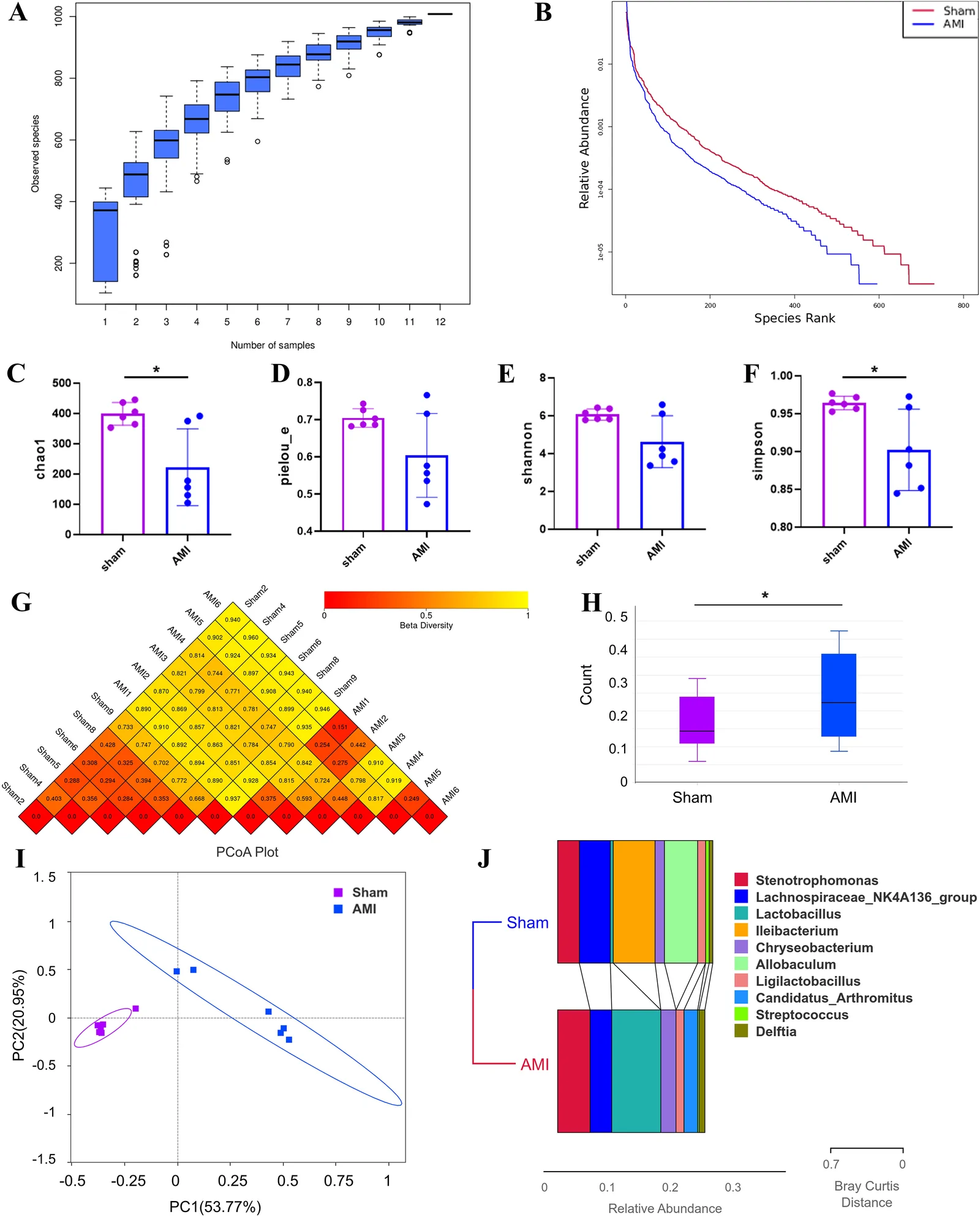
The α-diversity and β-diversity indices of gut microbiota. **(A-D)** The α-diversity indices (Chao1, Pielou_e, Shannon, Simpson) of gut microbiota in the AMI and Sham groups of mice; **(E)** The species accumulation boxplots for the AMI and Sham groups of mice; **(F)** The rank abundance curves for the AMI and Sham groups of mice; **(G)** Heatmaps of the Bray-Curtis distance matrices for the AMI and Sham groups of mice; **(H)** Boxplots of the β-diversity index distribution between the AMI and Sham groups of mice; **(I)** The β-diversity Principal Coordinate Analysis (PCoA) based on the Bray-Curtis distance for the AMI and Sham groups of mice; **(J)** UPGMA clustering tree at the genus level for the AMI and Sham groups of mice. T-test and Wilcoxon rank-sum test were performed between the two groups. *p < 0.05 vs. Sham; 6 mice per group; Error bars represent standard deviation.

Analysis of α-diversity and β-diversity is used to determine the richness and diversity of the overall microbial community and determine changes in the overall structure of gut microbiota because of AMI (Li et al., 2013). The species accumulation box plot was flat, thereby indicating that the sample sizes of the AMI and Sham group of mice were sufficient for further analysis (**Figure 3A**). The Rank abundance curve shows that the species richness was higher in the Sham group than in the AMI group, whereas species distribution was more uniform (**Figure 3B**). Compared with the Sham group, the Chao1 (Cohen’s d = -1.02, 95%CI: -1.95 ~ -0.09) and Simpson (Cohen’s d = -0.96, 95%CI: -1.88 ~ -0.04) indices of the AMI group were significantly decreased, whereas the Pielou_e and Shannon indices were decreased but without statistically significant differences (**Figures 3C–F**). These results confirm that the α-diversity of gut microbiota was significantly reduced in the AMI group, and the diversity and uniformity of the microbiota composition were impaired.

The Bray-Curtis distance (0–1, higher values indicate greater structural divergence) between samples was calculated using the evolutionary information from the microbial sequences in each sample, and a distance matrix was constructed. We found that there were significant inter-group differences in the gut microbiota community composition between mice in the Sham group and the AMI group (Bray-Curtis distance > 0.7), while the intra-group samples of each group showed high similarity in microbiota composition (**Figure 3G**). The inter-group difference test of the Beta index demonstrated significant differences in the microbial composition between the AMI and Sham groups (Adonis: r² = 0.36, *p* = 0.008; Anosim: r = 0.71, *p* = 0.006) (**Figure 3H**). Furthermore, results from the Principal Coordinates Analysis (PCoA) (PC1 = 53.77%), Principal Component Analysis (PCA), and Non-Metric Multi-Dimensional Scaling (NMDS) statistics (Stress < 0.1) based on the Bray-Curtis distance demonstrated distinct separation between the two groups (**Figure 3I**; **Supplementary Figures 1E, F**).

The Unweighted Pair Group Method with Arithmetic Mean (UPGMA) clustering tree analysis is a commonly used sample similarity clustering method. The UPGMA clustering trees at the phylum, genus, and species levels intuitively show the genetic relationship or grouping pattern of the microbiota between the two groups, thereby providing a key basis for analyzing differences in the microbial community structure. The UPGMA clustering tree analysis again demonstrated depletion of *I. valens, A. muciniphila*, and *T. pseudospiralis* in the AMI group compared with the Sham group at the species level. While Firmicutes decrease in the AMI group at phylum level, and lleibacterium, Allobaculum, and Streptococcus deplete in the AMI group, compared with the Sham group. (**Figure 3J**; **Supplementary Figures 1G, H**).

### 3.4 Statistical test for differences in gut microbiota community structure between the two groups of mice

**Figure 4:**
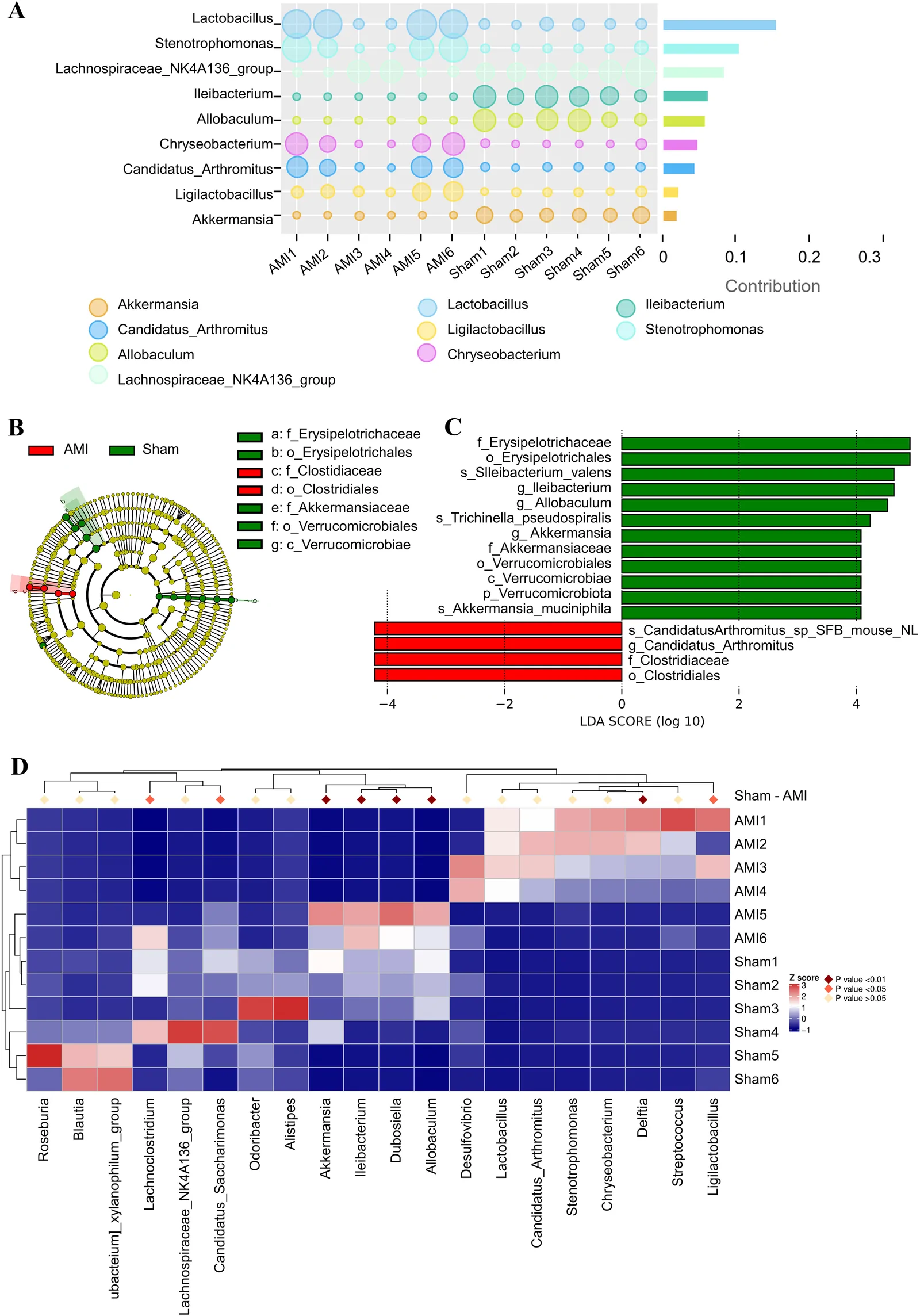
Statistical tests to evaluate differences in the gut microbiota community structure. **(A)** Simper bubble plot at the genus level for the AMI and Sham groups of mice; **(B)** LEfSe evolutionary branch diagram for the AMI and Sham groups of mice; **(C)** Bar chart of LDA value distribution for the AMI and Sham groups of mice; **(D)** MetagenomeSeq complex heatmap at the genus level for the AMI and Sham groups of mice.

SIMPER (Similarity Percentage) decomposes the Bray-Curtis dissimilarity index to reveal the contribution of each species towards the differences between the two groups. Simper bubble plots at the phylum, genus, and species levels show the top 10 phyla, genera, or species, respectively, which contribute to the differences between the two groups and their relative abundances (**Figure 4A**; **Supplementary Figures 2A, B**). At the genus level, *Lactobacillus* showed the highest relative abundance in the AMI group and contributed significantly to the differences between the two groups. *Akkermansia, Allobaculum*, and other genera also contributed to the differences between the two groups.

The LEfSe (LDA Effect Size) evolutionary cladogram (phylogenetic distribution) (**Figure 4B**) and LDA value distribution histogram (LDA > 4, *p* < 0.05, **Figure 4C**) showed that *Clostridiaceae* was the most dominant bacterial group in the AMI group mice, whereas *Erysipelotrichaceae, Akkermansiaceae*, and *Verrucomicrobiae* were the most dominant bacterial groups in the Sham group mice. The inter-group differential abundance t-test at the phylum, genus, and species levels identified species with significant inter-group differences at each taxonomic level (p < 0.05) and demonstrated a species evolutionary relationship between *Verrucomicrobiota*, *Akkermansia*, and *A. muciniphila* (**Supplementary Figures 2C–E**).

The MetagenomeSeq complex heatmap at the genus level showed significant inter-group differences in key genera such as *Akkermansia*, *Ileibacterium, Dubosiella, Allobaculum, Delftia* (*p* < 0.01), *Ligilactobacillus, Candidatus Saccharimonas, and Lachnoclostridium* (*p* < 0.05) (**Figure 4D**; **Supplementary Figures 3A–H**).

### 3.5 Dysregulation of microbial functional potential in AMI

**Figure 5:**
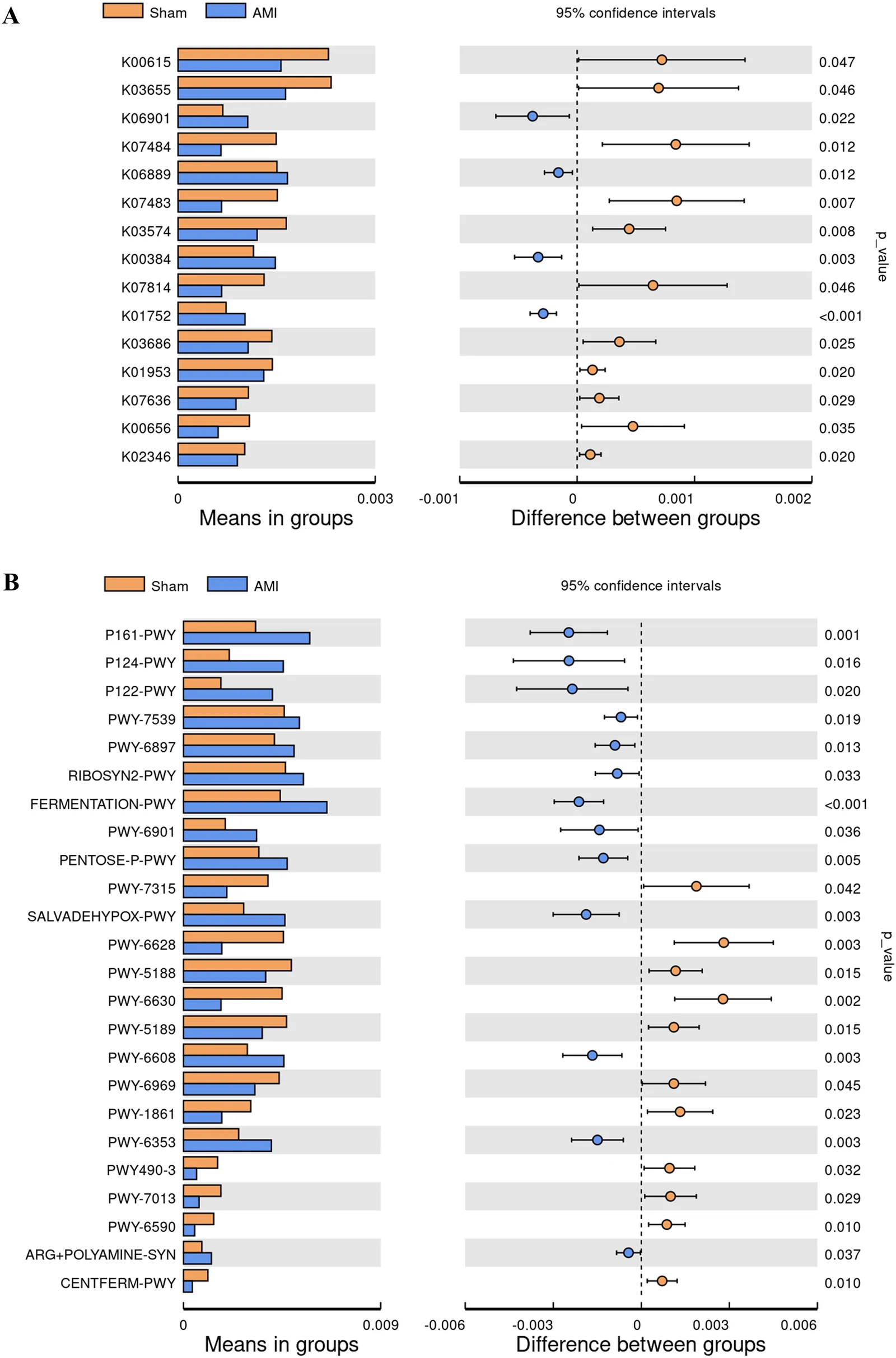
PICRUSt2 functional prediction of gut microbiota. **(A, B)** T-test of PICRUSt2 functional annotation of the gut microbiota based on the KEGG pathways between the AMI and Sham groups of mice. Adjusted p is shown in **Supplementary Data**.

Based on the KEGG (https://www.kegg.jp/) and MetaCyc (https://metacyc.org/) pathway databases, PICRUSt2 functional annotation tool was used to predict the metabolic functions of the top 10 genera with the highest abundance at the genus level (**Figures 5A, B**). The top differential pathways participate in the energy metabolism processes, including the tricarboxylic acid cycle and pentose phosphate pathway. These processes are critical in providing cells with reducing power, adapt to low-nutrient environments, and protect against oxidative stress. These mechanistic pathways predicted from the gut microbiota functions further demonstrate changes in the gut microbiota of AMI patients may affect the progression of the disease.

### 3.6 Quantitative differential analysis of gastrointestinal metabolites

**Figure 6:**
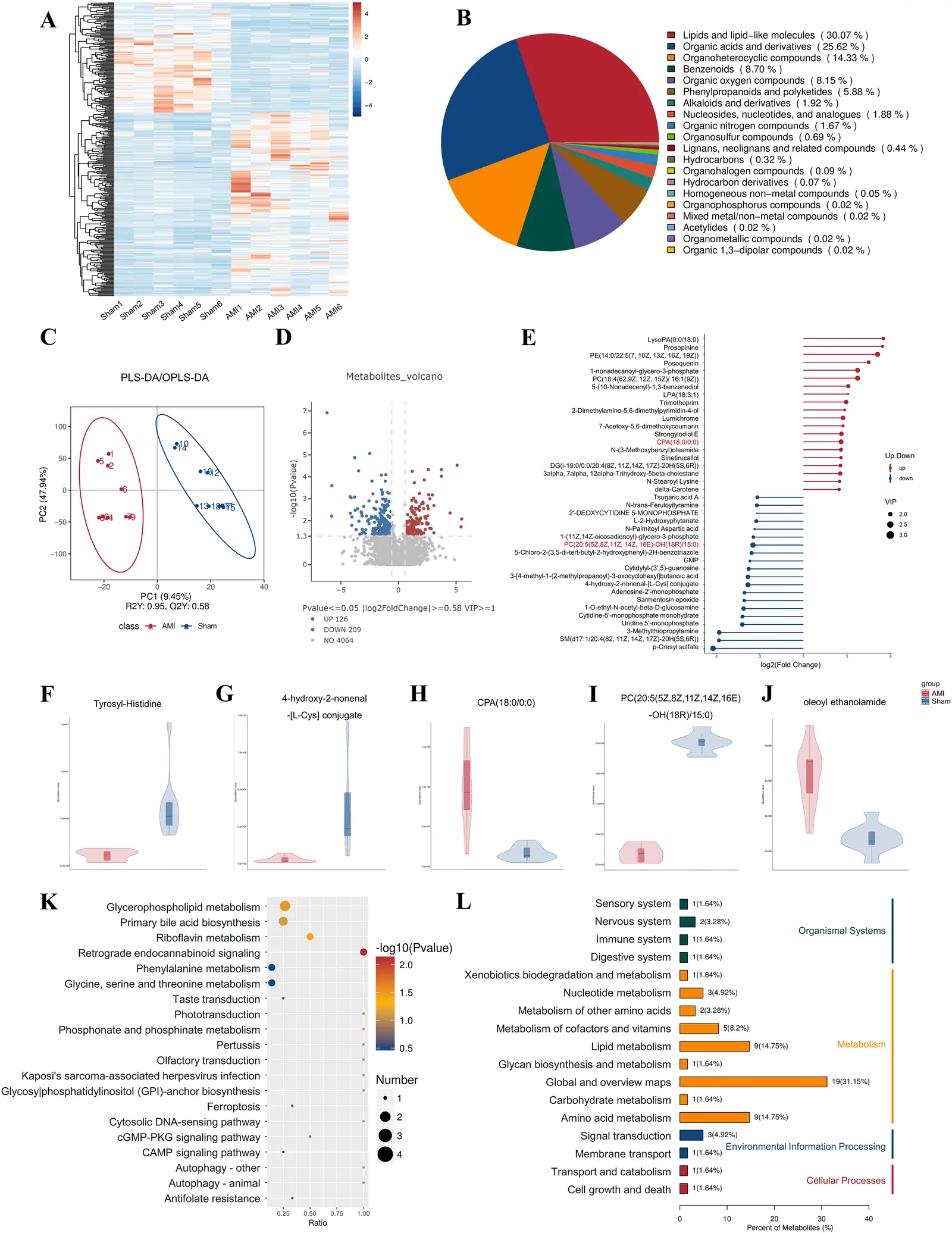
Quantitative analysis and pathway annotation of differential metabolites from gut intestinal contents. **(A)** Cluster analysis of differential metabolites; **(B)** Pie chart of metabolite Class I classification; **(C)** PLS-DA/OPLS-DA modeling results; **(D)** Volcano plot of differential metabolites; **(E)** Matchstick plot of differential metabolites; **(F-J)** Violin plots of differential metabolites; **(K, L)** Bubble chart **(K)** and bar chart **(L)** of KEGG pathway enrichment and annotation of differential metabolites.

We performed untargeted intestinal metabolomics of the AMI and Sham group mice and detected 4399 metabolites, including 1900 metabolites in the negative (neg) ion mode and 2499 metabolites in the positive (pos) ion mode (**Figure 6A**). According to Class I classification, the top 3 categories were lipids and lipid-like molecules (30.07%), organic acids and derivatives (25.62%) and organoheterocyclic compounds (14.33%) (**Figure 6B**).

Synergistic changes in multiple metabolites, rather than significant differences in individual metabolites are the foundation of metabolomics. Differential metabolites were screened mainly based on three parameters: VIP (Variable Importance in the Project) > 1.0, FC (fold change) > 1.5 or FC < 0.667, and *P-value* < 0.05. The noise from individual differences can obscure the synergistic signals and prevent clear group clustering in PCA, making it impossible to form obvious group clustering. However, PLS-DA/OPLS-DA (R2Y = 0.95 > Q2Y = 0.58) effectively integrates synergistic signals by superimposing the signals from multiple metabolites related to a distinct group through the partial least squares algorithm (**Figure 6C**). Combined with microbial data, our data shows that microbiota and metabolites coordinately regulates the progression of AMI.

As shown in the volcano plot (**Figure 6D**), compared with the Sham group, 126 metabolites are upregulated and 209 metabolites are downregulated in the AMI group. The matchstick plot (**Figure 6E**) shows the top 20 upregulated and downregulated metabolites. The chord diagram visually reflects the correlation and association degree between differential metabolites in the samples (**Supplementary Figure 4A**). The Pearson correlation coefficient between each pair of metabolites was calculated to specifically quantify the correlation, with absolute values closer to 1 indicating higher association degree between metabolites (**Supplementary Figure 4B**). We screened 5 differential metabolites related to nitrogen/aromatic metabolites, phospholipid metabolites, and oxidative stress-related metabolites and plotted violin plots to show their significantly different data distribution and probability density in the AMI and the Sham groups (**Figures 6F–J**).

The KEGG pathway database (https://www.genome.jp/kegg/pathway.html) classifies biological metabolic pathways into three levels. The three top enriched key differential pathways were retrograde endocannabinoid signaling (*p* = 0.0071), glycerophospholipid metabolism (*p* = 0.0311), and riboflavin metabolism (*p* = 0.0382) (**Figure 6K**). Our data showed that lipid metabolism (14.75%) and amino acid metabolism (14.75%) were the most enriched metabolic pathways (**Figure 6L**). Furthermore, Human Metabolome Database (HMDB) (https://hmdb.ca/metabolites) and LIPID MAPS (https://lipidmaps.org/) analyses annotated lipids and lipid-like molecules containing 663 metabolites and fatty acids and conjugates [FA01] containing 131 metabolites as the top-ranked pathways, respectively (**Supplementary Figures 4C, D**). This confirms that AMI-related metabolotypes were involved in lipid metabolism.

### 3.7 Analysis of differentially expressed genes and functional enrichment in myocardial tissues

**Figure 7:**
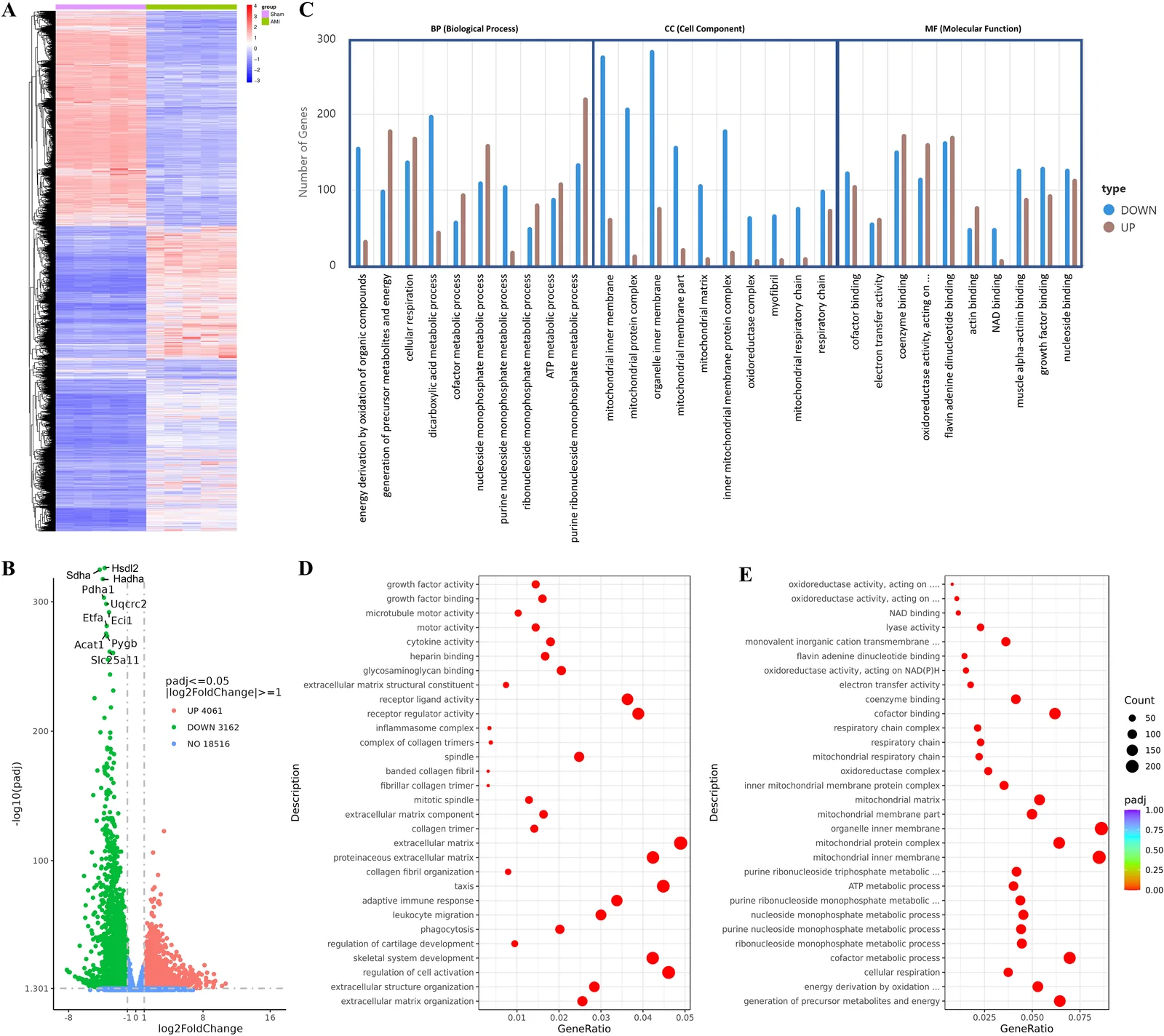
Quantitative analysis and pathway annotation of DEGs from damaged heart tissues. **(A)** Cluster analysis of differential genes; **(B)** Volcano plot of differential genes; **(C)** Gene up/down-regulation counts in the top 30 GO functional enrichment pathways; **(D, E)** Bubble plot of functional annotations for AMI up/down-regulated pathways.

Transcriptome sequencing was performed on myocardial tissues from mice in the AMI and Sham groups. The heatmap of differentially expressed genes (DEGs) revealed the overall distribution and expression variation trends of DEGs between the two groups (**Figure 7A**). The volcano plot intuitively showed 4061 significantly upregulated and 3162 significantly downregulated genes among DEGs (|log2FC|≥1, *padj* < 0.05), with the most prominent downregulation observed in key energy metabolism genes such as *Sdha, Hsdl2* and *Hadha* (**Figure 7B**).

Gene Ontology (GO) functional enrichment analysis demonstrated that the top 30 significantly enriched pathways covered three categories: biological process (BP), cellular component (CC) and molecular function (MF). The bar plot directly compared the enrichment numbers of upregulated and downregulated DEGs in each pathway (**Figure 7C**). Then, further functional enrichment analysis of upregulated and downregulated DEGs was conducted separately. The upregulated DEGs were significantly enriched in pathways including growth factor activity/binding, microtubule motor activity, extracellular matrix structural constituent, collagen fibril organization, adaptive immune response and phagocytosis, which reflected the pathological characteristics of stress repair, inflammatory response and extracellular matrix remodeling in myocardial tissues after AMI (**Figure 7D**); whereas the downregulated DEGs were highly concentrated in pathways such as oxidoreductase activity, NAD binding, mitochondrial respiratory chain complex, ATP metabolic process, cellular respiration and generation of precursor metabolites and energy. This directly uncovered the core phenotypic features of energy metabolism failure characterized by impaired mitochondrial structure and function and defective ATP production, which was highly consistent with the gene expression results (**Figure 7E**).

### 3.8 Correlation analysis of differential gut microbiota and differential metabolites

**Figure 8:**
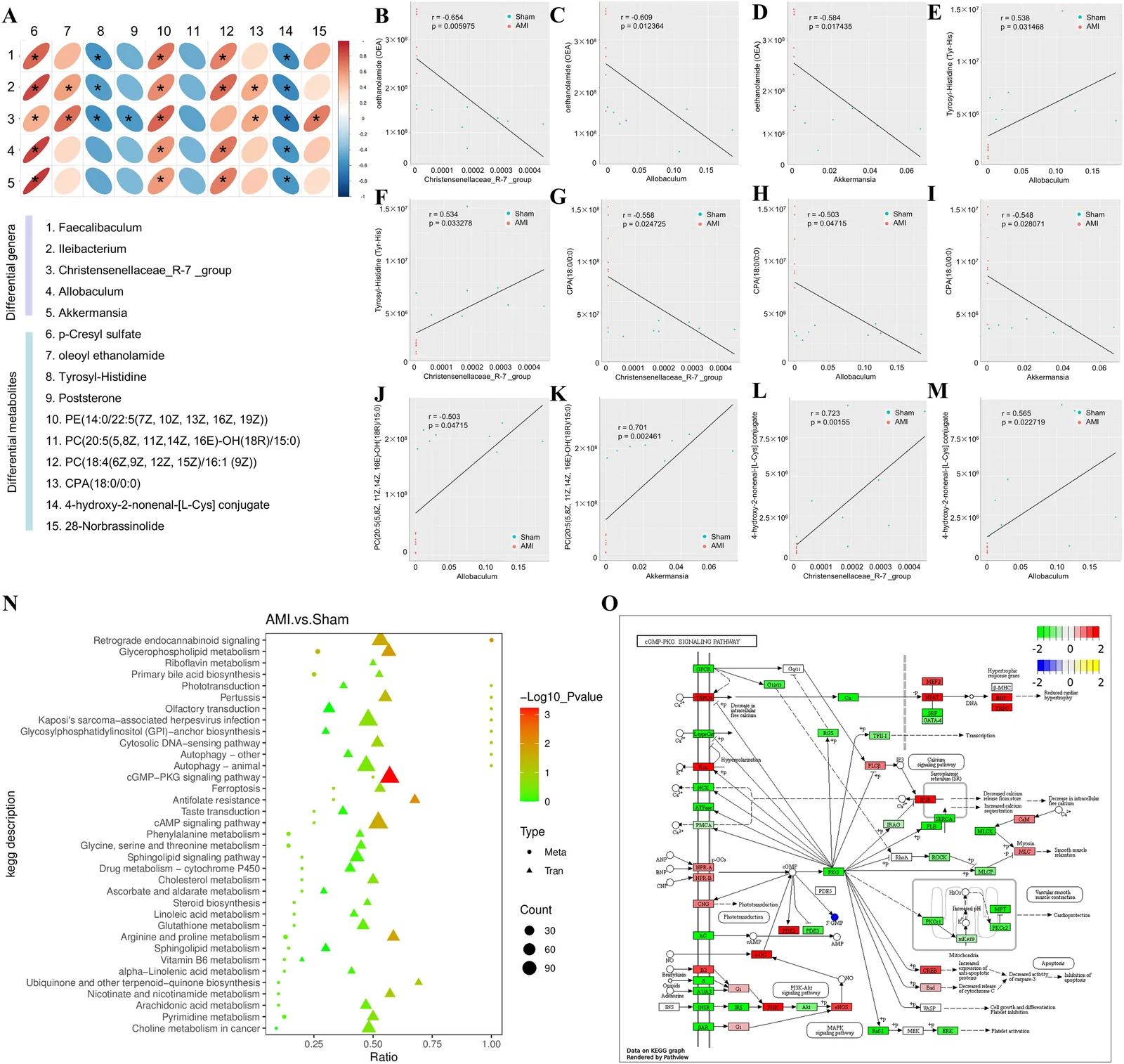
Correlation analysis of differential microbiota, metabolites and genes. **(A)** Heatmap for correlation analysis between differential gut microbiota and differential metabolites; **(B-M)** Scatter plot shows the top 12 pairs (largest |r| values) of differential genera and differential metabolites based on the Pearson correlation coefficient (r) and *P-value*. The value range of the correlation coefficient was (-1, 1). A correlation coefficient of less than 0 indicated negative correlation, whereas a correlation co-efficient greater than 0 indicated. The legend represents the correlation coefficient, with red indicating positive correlation and blue indicating negative correlation; the asterisk (*) mark indicates statistical significance; **(N)** Bubble plot of KEGG enrichment annotation for co-enriched pathways. Red indicates stronger significance; triangles represent enrichment of DEGs, dots represent enrichment of differential metabolites, and the shape size corresponds to the count of enriched genes/metabolites; **(O)** Pathview visualization of the cGMP-PKG signaling pathway. Green nodes represent downregulated expression, and red nodes represent upregulated expression.

Taking gastrointestinal metabolites as the bridge, we performed an integrated multi-omics correlation analysis. Pearson correlation analysis was first conducted between the top 5 differentially abundant genera (16S rRNA sequencing, genus level) and the top 15 differential metabolites (metabolomic analysis) (**Figure 8A**), and further performed between the top 50 DEGs (transcriptomic sequencing) and the top 50 differential metabolites (metabolomic analysis) (**Supplementary Figure 5**).

Scatter plot analysis of the correlation between metabolites and microbial flora with practical significance were performed based on the Pearson correlation coefficient (r) and p_value between differential genera and differential metabolites (**Figures 8B–M**). Our data showed that all three genera, *Christensenellaceae_R-7_group*, *Allobaculum*, and *Akkermansia* were downregulated in the AMI group. Furthermore, *Christensenellaceae_R-7_group* showed negative correlation with oleoyl ethanolamide (OEA) and CPA (18:0/0:0), and positive correlation with Tyrosyl-Histidine (Tyr-His) and 4-hydroxy-2-nonenal-[L-Cys] conjugate. *Allobaculum* showed negative correlation with OEA and CPA (18:0/0:0), and positive correlation with Tyr-His, PC (20:5(5,8Z,11Z,14Z,16E)-OH(18R)/15:0), and 4-hydroxy-2-nonenal-[L-Cys] conjugate. *Akkermansia* showed negative correlation with OEA and CPA (18:0/0:0), and positive correlation with PC (20:5(5,8Z,11Z,14Z,16E)-OH(18R)/15:0).

Based on the integrated transcriptome-metabolome association analysis, KEGG enrichment annotation was conducted for the key co-enriched pathways (**Figure 8N**). The top three predominant co-enriched pathways were identified as cGMP-PKG signaling pathway, retrograde endocannabinoid signaling, and autophagy-animal pathway. Furthermore, multiple lipid metabolism-related pathways, including glycerophospholipid metabolism, riboflavin metabolism, and primary bile acid biosynthesis, were also significantly enriched, highlighting the critical role of lipid metabolic dysregulation in the pathological progression of AMI.

## 4 Discussion

This multi-omics correlative study mapped gut microbial, metabolic and myocardial transcript shifts post-AMI. In previous studies (Li et al., 2025), we found that AMI mice exhibited digestive system symptoms, including significant changes in food intake and unformed feces (Umemura et al., 2024). This suggested that gastrointestinal health was strongly associated with cardiovascular health. Though our surgical model establishes myocardial injury as the primary initiating event, with gut microbial and metabolic shifts occurring as secondary downstream alterations, gut microbiota and metabolites may be involved in the regulation of cardiac function and associated with the physiological and pathological process of AMI. All results merely show associations rather than causality.

The first core correlative characteristic of the present study is intestinal microecological dysbiosis secondary to AMI-induced myocardial damage. Significant alterations in the overall structure and diversity of gut microbiota were observed in mice at day 7 after AMI modeling. Multiple short-chain fatty acid (SCFA), producing beneficial genera, including Akkermansia, Allobaculum, Christensenellaceae_R-7_group, and Ligilactobacillus, were markedly depleted in the AMI group, with Akkermansia showing the most dramatic reduction. These bacterial taxa are well recognized as essential sources of intestinal acetate and butyrate, and decreased SCFA abundance has been previously correlated with disordered myocardial energy metabolism, impaired mitochondrial β-oxidation, and increased cardiovascular disease risk (Carley et al., 2021). Consistently, the depletion of Akkermansia is associated with abnormal macrophage polarization, myocardial pyroptosis, and cardiac structural remodeling (Liu et al., 2025); altered abundance of Allobaculum is correlated with intestinal barrier dysfunction, myocardial hypertrophy, and cardiac fibrosis (Toya et al., 2020; Zhang et al., 2020); and reduced Christensenellaceae_R-7_group is closely linked to mitochondrial dysfunction and excessive inflammatory activation (Cui et al., 2023; Tannous et al., 2021). Functional enrichment analysis further indicated that perturbations of these differential genera were highly correlated with oxidative stress, inflammatory responses, and metabolic disorders, supporting a coordinated gut-cardiac phenotypic linkage following AMI.

The second core correlative axis focuses on the association between gut-derived metabolic disturbances and post-infarction myocardial lipid and energy metabolic dysfunction. Multi-omics profiling revealed extensive reprogramming of intestinal metabolites in AMI mice, characterized by disrupted lipid metabolism and activated oxidative stress pathways. The 4-hydroxy-2-nonenal-L-cysteine conjugate, a representative gut-derived oxidative stress metabolite, was differentially expressed in the AMI group. This metabolite is known to correlate with intestinal barrier disruption, ROS accumulation, and NLRP3 inflammasome activation (Li et al., 2019; Chancharoenthana et al., 2023), and its alteration covaried with myocardial inflammation, cardiomyocyte apoptosis, and cardiac dysfunction in the current AMI model. In addition, primary bile acid biosynthesis was identified as a key differential metabolic pathway. As direct metabolic products of gut microbiota, dysregulated bile acid metabolism is correlated with abnormal cardiac conduction and reduced myocardial contractility through FXR signaling (Bishop-Bailey et al., 2004; Mayerhofer et al., 2017).

Lipid bioactive molecules, amino acid derivatives, and phospholipid metabolites collectively constituted the characteristic metabolic signature associated with AMI pathology. Oleoylethanolamide (OEA), an endocannabinoid-related lipid amide, exhibits context-dependent dual biological effects in different disease models: it may alleviate ventricular remodeling via Ras-Erk signaling (Comella et al., 2024), while excessive OEA accumulation potentially triggers PPAR-α-mediated lipotoxicity and cardiomyocyte apoptosis (Rajlic et al., 2022). The aberrant OEA enrichment observed in AMI mice was closely correlated with myocardial injury phenotypes, though its exact functional role remains to be validated. Tyrosyl-histidine and its upstream amino acids, which are reportedly involved in cardiomyocyte survival, displayed distinct differential expression in the AMI metabolic disorder microenvironment. Moreover, phospholipid metabolites including PC and CPA were significantly altered under AMI ischemia-hypoxia and ATP depletion (Leibundgut et al., 2012). Perturbations of these phospholipids were correlated with both vascular inflammatory injury and antioxidant responses, showing ambiguous dual effects and representing potential AMI-associated metabolic biomarkers. Notably, no significant alterations in TMAO-related metabolites were detected in the present study, which is consistent with previous AMI studies using standard chow diets without high-protein or high-fat dietary intervention (Henein et al., 2022; Yin et al., 2015; Bjornestad et al., 2020). This observation confirms that dietary composition is a critical extrinsic regulator of intestinal TMAO biosynthesis and explains the specific metabolic characteristics of the current model (Wang et al., 2015).

The third and integrative core axis was established through multi-omics joint analysis, which identified three pivotal hub pathways that bridge altered gut microbiota, differential intestinal metabolites, and myocardial transcriptional reprogramming. The retrograde endocannabinoid signaling, glycerophospholipid metabolism, and riboflavin metabolism pathways served as unified regulatory frameworks underlying gut-heart crosstalk. All previously described differential bacterial genera (Akkermansia, Allobaculum, Christensenellaceae_R-7_group) and signature metabolites (bile acids, OEA, oxidative stress-related metabolites, and phospholipids) converged on these three hub pathways, which further matched the perturbed myocardial tricarboxylic acid cycle, pyruvate metabolism, and pentose phosphate pathways in AMI. Pathway visualization (**Figure 8O**) of cGMP-PKG signaling further illustrated that differential expression of core pathway molecules (NO, cGMP, PKG, IP_3_) and downstream effectors (MAPK, ERK, Bcl-2) was coordinately associated with altered myocardial calcium homeostasis, cardiomyocyte apoptosis, vasomotor regulation, and cytoprotective responses, providing intuitive multi-omics correlative evidence for post-AMI gut-cardiac metabolic interaction.

Global metabolic network analysis (**Supplementary Figure 6**) confirmed that AMI pathology was primarily characterized by lipid metabolic disturbance, myocardial energy exhaustion, and systematic metabolic reprogramming. Concurrent enrichment of xenobiotic metabolism and glycan metabolism further indicated that host-microbe metabolic crosstalk is extensively involved in AMI pathological progression. Importantly, discrete alterations in individual bacterial taxa and single metabolites did not function independently but were integrated components of the unified gut-heart regulatory network. This integrative interpretation resolves the inconsistency between individual omics variations and global multi-omics signatures and constructs a coherent correlative model of post-AMI intestinal and cardiac remodeling.

This study applied multi-omics correlation analysis to explore the changes in gut microbiota and metabolites associated with AMI. However, it has several limitations. First, the relatively small sample size, which is a common challenge in animal studies but may limit statistical power and influence robustness and reproducibility of the findings, particularly for high-dimensional microbiome and metabolomics analyses. While differences between groups are observed, it would reflect the secondary effects of surgery, stress, or altered feeding behavior. In addition to limited sample size, the observational research design cannot distinguish causal versus correlative relationships among gut microbiota, metabolites and myocardial phenotypes. All mechanistic hypotheses proposed herein are exploratory and require causal verification experiments, including fecal microbiota transplantation, probiotic intervention, metabolite supplementation, or gene knockout assays, to validate the direct regulatory effects of gut microbiota and metabolites on myocardial injury. Second, only male mice and the 7-day time point after left anterior descending coronary artery ligation was examined, which restricted the investigation of the disease progression and dynamic monitoring of changes in the gut microbiota and metabolites in different genders. In the future, we aim to further evaluate the therapeutic effects of cardiovascular drugs or prebiotics on the AMI mice and verify the accuracy of the differential metabolites identified and the regulatory and protective role of gut microbiota in the cardiovascular diseases (Zhou et al., 2025). Future longitudinal and gender controlled studies with multiple time points might be conducted to explore the underlying mechanism such as higher levels of inflammation, increased oxidative stress (Ahlawat et al., 2021), especially excess production of reactive oxygen species (ROS) associated with persistent inflammation (Curtis et al., 2018).

## 5 Conclusions

We found significant correlations of the gut microbiota, metabolites, and other factors with the pathogenesis of AMI in our study. This suggests that the gastrointestinal tract and cardiovascular system may be closely interconnected via the microbiota-metabolism-inflammation network, providing a new paradigm of treating the heart from the gut for precision medicine and new scientific clues for clinical comorbidity management, comprehensive treatment and the development of precision therapeutic strategies, laying a foundation for future confirmatory experiments.

## Data Availability

The datasets presented in this study can be found in online repositories. The names of the repository/repositories and accession number(s) can be found in the article/**Supplementary Material**.
