# Supplementary material for: Gastrointestinal metabolites: a potential bridge connecting gut microbiota and myocardial metabolic reprogramming in acute myocardial infarction
Source: Front Physiol. 2026 Aug 17;17:1837224. doi: 10.3389/fphys.2026.1837224 (PMC13526554; doi:10.3389/fphys.2026.1837224)
Supplement: Supplementary file 1 [file SupplementaryFile1.docx]

**Supplementary Material**

**Supplementary Figure 1. Mice in the AMI group exhibit alterations in the gut microbiota.**

(A-B) Circos plots show the relationships between samples and gut microbiota at the phylum and species levels. (C-D) Clustering heatmaps show gut microbiota abundance at the phylum and species levels. (E) Principal Component Analysis (PCA); (F) Non-metric Multidimensional Scaling (NMDS); (G-H) UPGMA clustering trees at the phylum and species levels.

**Supplementary Figure S2. Intergroup differences in the gut microbiota community structure**

Simper analysis (A) at phylum level; (B) at species level. Differential gut bacterial taxa (C) at phylum level; (D) at genus level; (E) at species level.

**Supplementary Figure S3. Metagenome sequencing-based analysis of intergroup differences in the gut microbiota at the genus level**

(A) Ligilactobacillus; (B) Delftia; (C) Ileibacterium; (D) Allobaculum; (E) Dubosiella; (F) Akkermansia; (G) Lachnoclostridium; (H) Candidatus_Saccharimonas.

**Supplementary Figure S4.** **Quantitative differential analysis and pathway annotation of metabolomics from gutintestinal contents**

(A) Chord diagram of differential metabolites; (B) Pearson correlation between differential metabolites; (C) HMDB classification annotation map; (D) LIPID MAPS classification annotation map.

**Supplementary Figure S5. Heatmap for correlation analysis between DEGs and differential metabolites**

**Supplementary Figure S6. Integrated visualization of the global metabolic pathway network**
